# Supplementary figures and images for: Effect of virtual reality-simulated exercise on sympathovagal balance
Source: PLoS One. 2020 Jul 16;15(7):e0235792. doi: 10.1371/journal.pone.0235792 (PMC7365438; doi:10.1371/journal.pone.0235792)

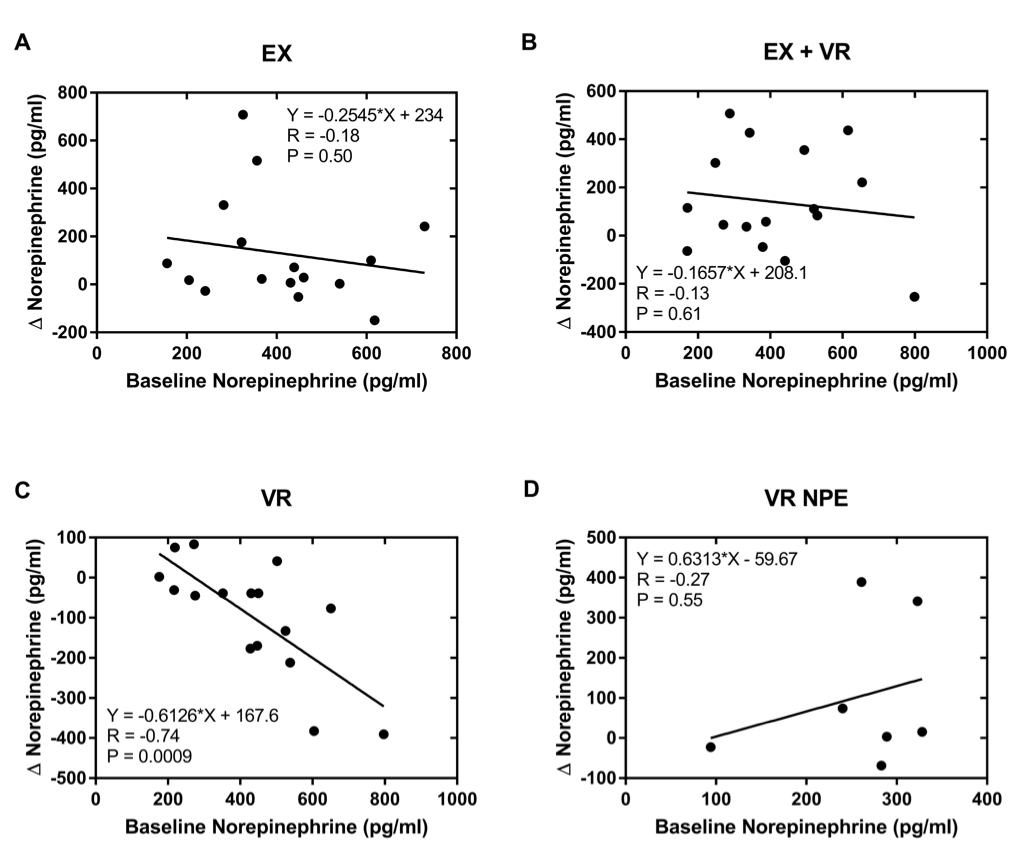

Supplement: S1 Fig — A, Correlation in cycling group; B, Correlation for group that cycled using the VR environment; C, Correlation for group that experienced virtual exercise without cycling; D, Correlation for group that experienced vitual exercise without prior exposure. (TIF) [file pone.0235792.s001.tif]
