# Supplementary material for: Effect of virtual reality-simulated exercise on sympathovagal balance
Source: PLoS One. 2020 Jul 16;15(7):e0235792. doi: 10.1371/journal.pone.0235792 (PMC7365438; doi:10.1371/journal.pone.0235792)
Supplement: S1 Table — (DOCX) [file pone.0235792.s002.docx]

**S1 Table: Participants’ characteristics, hemodynamic variables and norepinephrine levels pre- and post-exercise as mean +/- SD.** HI = high-intensity exerciser. MI = moderate-intensity exerciser. NPE = VR exposure with no prior exercise exposure. Units are as follows: Age – years. Systolic – mmHg. Diastolic – mmHg. Heartrate – beats per minute. Norepinephrine = pg/ml.

| Group | Age | Session | Pre - Exercise | | | | Post - Exercise | | | |  |
| --- | --- | --- | --- | --- | --- | --- | --- | --- | --- | --- | --- |
|  |  |  | Systolic | Diastolic | HR | NE | Systolic | Diastolic | HR | NE | % Max HR |
| HI | 22.5 +/- 2.07 | 1 | 114 +/-17.08 | 71.16 +/- 11.44 | 78.5 +/- 8.66 | 476.33 +/- 145.83 | 133.5 +/- 28.48 | 79.16 +/- 14.27 | 118 +/- 10.05 | 717.33 +/-  273.38 | 80.90 +/- 6.86 |
|  |  | 2 | 109.66 +/- 16.89 | 70.66 +/- 10.78 | 77 +/- 16.09 | 454.66 +/- 156.07 | 125.83 +/-27.01 | 80.16 +/-11.46 | 114.66 +/- 8.52 | 785.33 +/-225.92 |  |
|  |  | 3 | 107.66 +/- 12.65 | 69 +/- 5.62 | 81.5 +/-12.43 | 453 +/- 175.92 | 113.16 +/-11.63 | 78.33 +/- 10.13 | 82 +/- 13.40 | 352.16 +/- 124.13 |  |
| MI | 21.63 +/- 1.20 | 1 | 123 +/- 22.29 | 75.09 +/- 8.99 | 70.72 +/- 8.77 | 367.1 +/- 150.73 | 130.72 +/- 17.75 | 82.27 +/- 11.61 | 92.63 +/- 15.04 | 430.8 +/- 158.15 | 60.02 +/- 8.53 |
|  |  | 2 | 119 +/- 18.60 | 77.72 +/- 14.12 | 66.27 +/- 9.10 | 384.18 +/- 207.62 | 126 +/- 10.43 | 81.09 +/- 12.24 | 93.54 +/- 17.59 | 407 +/- 176.50 |  |
|  |  | 3 | 115.54 +/- 15.73 | 74.90 +/- 9.37 | 71.54 +/- 10.90 | 416.5 +/- 179.54 | 118.63 +/- 19.56 | 79.72 +/- 14.77 | 74.72 +/- 8.37 | 323.5 +/- 116.13 |  |
| NPE | 21.28 +/- 1.38 | NA | 109.57 +/- 19.73 | 70.14 +/- 11.29 | 70.14 +/- 9.02 | 259.71 +/- 79.49 | 113.28 +/- 17.73 | 83 +/-11.56 | 77.71 +/- 10.79 | 364 +/- 219.19 | NA |
