# Supplementary material for: Effect of virtual reality-simulated exercise on sympathovagal balance
Source: PLoS One. 2020 Jul 16;15(7):e0235792. doi: 10.1371/journal.pone.0235792 (PMC7365438; doi:10.1371/journal.pone.0235792)
Supplement: S2 Table — (DOCX) [file pone.0235792.s003.docx]

**S2 Table: Participants’ characteristics, hemodynamic variables and norepinephrine levels pre- and post-exercise.** HI = high-intensity exerciser. MI = moderate-intensity exerciser. NPE = VR exposure with no prior exercise exposure. Units are as follows: Age – years. Systolic – mmHg. Diastolic – mmHg. Heartrate – beats per minute. Norepinephrine = pg/ml.

|  | | | | Pre - Exercise | | | | Post - Exercise | | | |  |
| --- | --- | --- | --- | --- | --- | --- | --- | --- | --- | --- | --- | --- |
| Participant | Group | Age | Session | Systolic | Diastolic | Heartrate | Norepinephrine | Systolic | Diastolic | Heartrate | Norepinephrine | % Max Heartrate |
| 1 | HI | 24 | 1 | 118 | 77 | 62 | 325 | 169 | 90 | 127 | 1033 | 82.45 |
|  |  |  | 2 | 119 | 66 | 57 | 334 | 141 | 76 | 103 | 371 |  |
|  |  |  | 3 | 118 | 69 | 62 | 604 | 123 | 84 | 61 | 221 |  |
| 2 | HI | 26 | 1 | 119 | 74 | 82 | 356 | 165 | 89 | 108 | 872 | 70.62 |
|  |  |  | 2 | 127 | 73 | 72 | 494 | 147 | 98 | 117 | 849 |  |
|  |  |  | 3 | 117 | 73 | 83 | 525 | 123 | 90 | 90 | 392 |  |
| 3 | HI | 21 | 1 | 115 | 54 | 84 | 460 | 121 | 56 | 124 | 489 | 84.71 |
|  |  |  | 2 | 99 | 56 | 83 | 343 | 129 | 78 | 117 | 770 |  |
|  |  |  | 3 | 100 | 64 | 100 | 219 | 119 | 73 | 95 | 294 |  |
| 4 | HI | 22 | 1 | 141 | 87 | 80 | 540 | 136 | 93 | 130 | 543 | 77.57 |
|  |  |  | 2 | 128 | 89 | 90 | 288 | 153 | 89 | 127 | 795 |  |
|  |  |  | 3 | 121 | 76 | 79 | 651 | 117 | 85 | 90 | 574 |  |
| 5 | HI | 21 | 1 | 94 | 63 | 77 | 729 | 102 | 71 | 109 | 971 | 90.87 |
|  |  |  | 2 | 93 | 69 | 62 | 615 | 92 | 66 | 107 | 1052 |  |
|  |  |  | 3 | 90 | 61 | 78 | 447 | 100 | 62 | 70 | 277 |  |
| 6 | HI | 21 | 1 | 97 | 72 | 86 | 448 | 108 | 76 | 110 | 396 | 79.20 |
|  |  |  | 2 | 92 | 71 | 98 | 654 | 93 | 74 | 117 | 875 |  |
|  |  |  | 3 | 100 | 71 | 87 | 272 | 97 | 76 | 86 | 355 |  |
| 7 | MI | 21 | 1 | 96 | 65 | 74 | 431 | 121 | 74 | 93 | 438 | 66.87 |
|  |  |  | 2 | 97 | 57 | 73 | 270 | 104 | 69 | 88 | 315 |  |
|  |  |  | 3 | 94 | 67 | 72 | 176 | 105 | 66 | 77 | 178 |  |
| 8 | MI | 21 | 1 | 103 | 68 | 60 | 439 | 125 | 79 | 75 | 510 | 54.07 |
|  |  |  | 2 | 98 | 61 | 50 | 248 | 126 | 67 | 76 | 550 |  |
|  |  |  | 3 | 105 | 73 | 58 | 797 | 115 | 72 | 61 | 406 |  |
| 9 | MI | 23 | 1 | 112 | 76 | 67 | 156 | 126 | 79 | 114 | 244 | 40.58 |
|  |  |  | 2 | 109 | 75 | 73 | 441 | 113 | 85 | 102 | 336 |  |
|  |  |  | 3 | 105 | 75 | 74 | 275 | 94 | 71 | 87 | 230 |  |
| 10 | MI | 21 | 1 | 162 | 88 | 82 | 322 | 169 | 103 | 100 | 498 | 62.76 |
|  |  |  | 2 | 138 | 81 | 66 | 388 | 132 | 88 | 99 | 446 |  |
|  |  |  | 3 | 145 | 81 | 81 | 538 | 132 | 83 | 83 | 326 |  |
| 11 | MI | 23 | 1 | 115 | 80 | 72 | 205 | 119 | 85 | 111 | 223 | 68.78 |
|  |  |  | 2 | 125 | 89 | 75 | 170 | 140 | 83 | 118 | 106 |  |
|  |  |  | 3 | 116 | 88 | 94 | 217 | 113 | 87 | 76 | 186 |  |
| 12 | MI | 21 | 1 | 143 | 83 | 72 | 610 | 130 | 75 | 96 | 710 | 60.01 |
|  |  |  | 2 | 139 | 98 | 64 | 799 | 126 | 84 | 82 | 545 |  |
|  |  |  | 3 | 121 | 80 | 70 | 502 | 114 | 81 | 79 | 543 |  |
| 13 | MI | 24 | 1 | 118 | 64 | 81 | 282 | 135 | 77 | 106 | 613 | 68.76 |
|  |  |  | 2 | 114 | 72 | 70 | 521 | 119 | 75 | 102 | 632 |  |
|  |  |  | 3 | 118 | 61 | 71 | 450 | 119 | 65 | 76 | 411 |  |
| 14 | MI | 20 | 1 | 132 | 88 | 74 | 241 | 139 | 97 | 73 | 214 | 55.95 |
|  |  |  | 2 | 130 | 85 | 66 | 171 | 135 | 92 | 80 | 286 |  |
|  |  |  | 3 | 126 | 86 | 68 | 352 | 157 | 106 | 71 | 313 |  |
| 15 | MI | 21 | 1 | 123 | 69 | 59 | 367 | 138 | 68 | 68 | 390 | 58.51 |
|  |  |  | 2 | 109 | 65 | 52 | 379 | 126 | 65 | 72 | 332 |  |
|  |  |  | 3 | 119 | 65 | 52 | 428 | 112 | 68 | 59 | 251 |  |
| 16 | MI | 22 | 1 | 153 | 81 | 56 | NA | 143 | 99 | 100 | NA | 63.99 |
|  |  |  | 2 | 150 | 99 | 65 | NA | 137 | 107 | 79 | NA |  |
|  |  |  | 3 | 130 | 83 | 73 | NA | 147 | 106 | 77 | NA |  |
| 17 | MI | 21 | 1 | 96 | 64 | 81 | 618 | 93 | 69 | 83 | 468 | NA |
|  |  |  | 2 | 97 | 72 | 81 | 530 | 115 | 83 | 119 | 614 |  |
|  |  |  | 3 | 92 | 65 | 74 | 430 | 97 | 72 | 76 | 391 |  |
| 18 | NPE | 20 | NA | 115 | 73 | 72 | 323 | 125 | 85 | 80 | 664 | NA |
| 19 | NPE | 20 |  | 112 | 75 | 64 | 289 | 113 | 88 | 69 | 292 |  |
| 20 | NPE | 24 |  | 140 | 84 | 70 | 328 | 132 | 99 | 77 | 343 |  |
| 21 | NPE | 21 |  | 93 | 69 | 70 | 94 | 84 | 69 | 78 | 71 |  |
| 22 | NPE | 21 |  | 122 | 71 | 68 | 240 | 132 | 91 | 89 | 314 |  |
| 23 | NPE | 21 |  | 106 | 72 | 88 | 283 | 104 | 67 | 91 | 214 |  |
| 24 | NPE | 22 |  | 79 | 47 | 59 | 261 | 103 | 82 | 6 | 650 |  |
