# Supplementary material for: Effect of virtual reality-simulated exercise on sympathovagal balance
Source: PLoS One. 2020 Jul 16;15(7):e0235792. doi: 10.1371/journal.pone.0235792 (PMC7365438; doi:10.1371/journal.pone.0235792)
Supplement: Attachment — Submitted filename: response letter.docx [file pone.0235792.s004.docx]

**Response Letter**

We thank the reviewers for their thoughtful criticisms and encouraging comments. We have revised the manuscript to address reviewer concerns, and we provide a point-by-point response below, with reviewer comments in Times New Roman and our responses in Arial. Line numbers mentioned in this letter refer to the “redline” version.

Reviewer 1:

Major issues:
-In the abstract and even in the manuscript the authors mention sympathetic activity measured by heart rate variability. In the methods when the authors outline the physiological mechanisms behind the HRV measurements, none of them are listed as having SNS influence. The lone one would be LFnu, but in reality that is nothing more than 1-HFnu so if anything it is a vicarious measure of SNS outflow. If this is the case, then how do the authors justify HRV as measures of SNS outflow? On page 14, line 249 the authors state that LF/HF is a measure of SNS outflow, but that is not what they said on page 12, lines 188-89. This makes this paper really hard to follow, and confusing.

We agree with the reviewer’s point that HRV results should be interpreted with due caution. It is generally accepted that measures in the high frequency range of heart rate variability reflect modulation of parasympathetic nervous system activity. On the other hand, measures of the LF/HF ratio are more controversial. Changes in HF can lead to alterations in the LF/HF independent of modifications in LF. In our study this was clearly not the case, since as shown in Fig. 4, despite the similarity in HF between the HI and MI groups for VR exercise, a significant difference in LF/HF was evident. Likewise, even though HF was different between the HI and MI groups for EX, LF/HF was similar. Under these circumstances, we feel it is safe to assert that modifications in the LF/HF ratio reflect sympathovagal balance, and that our findings indicate differences in sympathovagal balance between the MI and HI groups during VR. We have revised the text to make this more clear in both places the reviewer mentioned, both on p.14, line 249 and on p.12, lines 188-189, as well as throughout the manuscript (these statements are highlighted in the redline version). Our text now makes explicit that our interpretation centers on sympathovagal balance and not on sympathetic outflow, per se.

-No hypothesis is provided. Without this vital bit of information it is impossible to know what the authors think will happen with the data in response to the intervention.

Hypothesis statement can now be found on lines 78-80, and reads “we hypothesized that simulated exercise may lead to increased norepinephrine release and modifications in autonomic control of heart rate during the period of stimulation.”

Minor issues:
-In the abstract, line 39, I read the word, 'caused.' I'd be cautious with this as the data you are presenting is nothing more than a correlate.

We removed the word “caused”. The sentence now says, “However, heart rate variability measured during virtual exercise was comparable to actual cycling in participants that engaged in moderate exercise…”.

-Page 3, first paragraph. From a writing perspective I would always say that a paragraph is 3 sentences, this one is two.

We combined the first two paragraphs, so that the resulting paragraph is now 5 sentences.

-Table 1 is very hard to follow. A lot of abbreviations and no caption. Based on its placement none of these things MI, HI or NPE have been introduced. Also, none of the variables have units.

We moved the Table into supplemental material, and added a caption that defines all the abbreviations in the table and the units for each measure. This is now Supplemental Table 2. We also added an additional Supplemental Table with average values for each group with standard deviations, and this is now Supplemental Table 1.

-Page 9, line 136. This method for calculating max HR needs a reference.

We have added the following reference for this calculation:  "Longitudinal Modeling of the Relationship between Age and Maximal Heart Rate", GELLISH, RONALD L.; GOSLIN, BRIAN R.; OLSON, RONALD E.; McDONALD, AUDRY; RUSSI, GARY D.; MOUDGIL, VIRINDER K. Medicine & Science in Sports & Exercise: May 2007 - Volume 39 - Issue 5 - p 822-829

-Page 10, line 159. What is the ICC for BP? No mention of reliability or validity.

We have added these measurements separately for the HI and MI groups in the methods section starting on line 156. “Intra-correlation coefficients within each group (Groups HI and MI are described below) were greater than or equal to 0.86 for systolic and 0.75 for diastolic, indicating a high degree of reproducibility between baseline measurements for each individual.”

-Page 12. No mention of what tests were run for normality, or what data were transformed. This limits the readers comprehension severely.

We have added this description on lines 191-192

-Results. Why was the decision made to only include p values? This is very limiting as that only addresses type I error, correct? Consider adding ES, CIs, etc, as measures of power.

We added E.S and C.I. to each stated p-value.

-References are often incomplete (some are missing the journal name) and they are inconsistent (journal name either missing caps, or is abbreviated).

Corrections have now been made to the references.

Reviewer #2: This article examined effects of VR simulation of exercise on sympathetic activity in stationary humans (vs VR exercise cycling). Main findings were that heart rate variability increase with VR exercise simulation, but there was no change in norepinephrine or heart rate with VR simulated exercise. The data is well presented, and paper well written. See minor comments below:

Abstract:
Line 29: Please abbreviate 'virtual reality' as "VR" starting here, and throughout the paper: "... to virtual reality (VR) simulation of exercise...

We have eliminated all use of “virtual reality” after the initial use, and replaced it with VR

Line 31: Change the word "bicycling" to "cycling" throughout, as this is a stationary cycle ergometer (not a true bicycle).

We have changed all mentions of bicycling to cycling

Line 33: Make it clear here that the "simulation" session included no actual exercise: "...to simulate exercise (without cycling exercise)..."

We added the words “without actual cycling exercise” here

Line: 34: Abbreviate 'heart rate' as "HR" starting here, and throughout the paper.

We changed all instances of “heart rate” to HR except in headings or when part of another phrase, such as heart rate variability.

Line 36: "find" should be "found"

We changed “find” to “found”

Introduction:

Line 48: Please specify this is "Chronic aerobic exercise" in the first sentence and combine the 1st and 2nd paragraphs

We added the word “chronic” and combined the first and second paragraphs

Line 53: Make it clear that these data are from the US: "...of the population of the United States (US) exercises regularly...”

We changed to read “United States population”

Line 58: Again, abbreviate 'virtual reality' as "VR" starting here, and throughout the paper.

We changed all instances of “virtual reality to VR”

Line 62: Specify that it is either a "head-mounted display or wall projector"

We removed “wall-mounted display to make it clear that head-mounted display was used in this study”

Line 86: Include this group at the end of the intro: "... with chronic disease or disability."

We added “chronic disease or disability”

Methods:
Table 1: Please report this table with individual data as a supplement table. The data for each participant is not necessary here. Instead please re-create this table with 'mean +/- SD in each group, session, and variable pre- and post- exercise (be sure to identify which session was which, and identify abbreviations in the heading (HI, MI, NPE).RJ
Line 111: I'm not sure that 2 hours without stimulants is long enough to clear from a human system. Please state why 2 hours was chosen as a cutoff for abstaining from caffeine and nicotine, and not longer. Also, where any other controls taking into account? Did the participants refrain from exercise prior to the trials? This could have affected baseline norepinephrine levels.

In retrospect, we should have asked for a longer time without stimulants and perhaps baseline results for NE would have been less variable. In follow-up studies, we plan to increase this mandated time, and attempt to better enforce the guidelines. We have recognized this in our discussion of limitations to the study.

Line 136: Please cite the source of this Max HR equation

Now cited, see above response to reviewer 1’s similar comment.

Line 154: Briefly describe your ELISA protocol (or cite source of similar protocol from your lab).

We have added description of ELISA protocol starting on line 148.

Line 182: Space out "minutesin"

We added the space

Line 209: State your alpha-level for significance and any statistical analysis software used.

We now state the significance threshold and software used for stats in the methods starting on line 204.

Discussion:
Line 284: In the discussion, I think it's also important to note that VR exer-gaming is also being used by healthy people for exercise, and has been shown to increase metabolic rate (measured by VO2 consumption) and HR. Metabolic rate would be a good measure for future studies on virtual exercise... Consider including a sentence like this:
"...particularly well-developed [32, 33]. Movement based VR exer-gaming is also becoming a popular exercise modality in health adults, and has been shown in increase HR and metabolic rate (oxygen consumption, VO2) from 'moderate' to 'vigorous' levels depending on the VR experience (Cite Gomez, 2018). However, the use of VR to induce...". Please cite Gomez 2018 here (<https://pubmed.ncbi.nlm.nih.gov/30325233/>).

The suggested sentence and citation have been added starting on line 293. Thanks for this suggestion.

References:
Please ensure that references are correctly formatted (some journal titles are capitalized, others are not).

We fixed the reference formatting.
