# Supplementary material for: Effect of virtual reality-simulated exercise on sympathovagal balance
Source: PLoS One. 2020 Jul 16;15(7):e0235792. doi: 10.1371/journal.pone.0235792 (PMC7365438; doi:10.1371/journal.pone.0235792)
Supplement: Attachment — Submitted filename: Response To Reviewers.docx [file pone.0235792.s005.docx]

**Response To Reviewers**

We thank the reviewers for their suggestions and criticisms which have substantially improved the manuscript. The reviewer comments are listed below in italics, with our responses in normal font.

*Page 4, line 78. The authors simply state, '...modifications in autonomic control...' as part of their hypothesis. This is pretty non-descriptive and doesn't really help the reader. I'd suggest being specific here.*

We changed the sentence to read as follows:

As a first step toward this goal, we hypothesized that simulated exercise may lead to increases in sympatho-vagal balance and decreases in parasympathetic nervous system activity that are ultimately coupled to increased norepinephrine release and heart rate.

*Page 12, line 250. The authors state '(HF)' but don't they really mean (lnHF)?*

We have changed this to say (lnHF).

*Page 12, line 266. Post exercise or post-exercise? The authors keep changing. I'd suggest double checking document for consistency.*

We have double-checked and the manuscript now consistently uses hyphens after each use of “post”.
